# Protein-protein interaction–interfering peptide rescues dysregulated NMDA receptor signaling

**DOI:** 10.1172/jci.insight.189634

**Published:** 2025-12-04

**Authors:** Robert E. Featherstone, Hongbin Li, Ameet S. Sengar, Karin E. Borgmann-Winter, Olya Melnychenko, Lindsey M. Crown, Ray L. Gifford, Felix Amirfathi, Anamika Banerjee, AiVi Tran, Krishna Parekh, Margaret Heller, Wenyu Zhang, Robert J. Gallop, Adam D. Marc, Pragya Komal, Michael W. Salter, Steven J. Siegel, Chang-Gyu Hahn

**Affiliations:** 1 Department of Psychiatry and the Behavioral Sciences, University of Southern California, Los Angeles, California, USA.; 2 Neurosciences and Mental Health Program at SickKids Research Institute Toronto, Ontario, Canada.; 3 Department of Physiology at the University of Toronto, Ontario, Canada.; 4 Department of Psychiatry and Behavioral Sciences & Neuroscience,; 5 Department of Neuroscience, and; 6 Vickie & Jack Farber Institute for Neuroscience, the Sidney Kimmel Medical College at Thomas Jefferson University, Philadelphia, Pennsylvania, USA.; 7 Research and Development, Rocket Pharmaceuticals, Cranbury, New Jersey, USA.; 8 Department of Otorhinolaryngology, University of Pennsylvania, Philadelphia, Pennsylvania, USA.; 9 Department of Mathematics, West Chester University, West Chester, Pennsylvania, USA.

**Keywords:** Cell biology, Neuroscience, Protein kinases, Psychiatric diseases, Schizophrenia

## Abstract

The complex and heterogeneous genetic architecture of neuropsychiatric illnesses compels us to look beyond individual risk genes for therapeutic strategies and target the interactive dynamics and convergence of their protein products. A mechanistic substrate for convergence of synaptic neuropsychiatric risk genes are protein-protein interactions (PPIs) in the N-methyl-D-aspartate receptor (NMDAR) complex. NMDAR hypofunction in schizophrenia is associated with hypoactivity of Src kinase, resulting from convergent alterations in PPIs of Src with its partners. Of these, the association of Src with PSD-95, which inhibits the activity of this kinase in the NMDAR complex, is known to be increased in schizophrenia. Here, we devised a strategy to suppress the inhibition of Src by PSD-95 by employing a cell-penetrating and Src-activating PSD-95 inhibitory peptide (TAT-SAPIP). TAT-SAPIP enhanced synaptic NMDAR currents in *Src^+/–^* and *Sdy^–/–^* mice manifesting NMDAR hypofunction phenotypes. Chronic intracerebroventricularly (ICV) injection of TAT-SAPIP rescued cognitive deficits in trace fear conditioning in *Src ^+/–^* mice. Moreover, TAT-SAPIP enhanced Src activity in synaptoneurosomes derived from dorsolateral prefrontal cortex of 14 patients. We propose blockade of the Src–PSD-95 interaction as a proof of concept for the use of interfering peptides as a therapeutic strategy to reverse NMDAR hypofunction in schizophrenia and other illnesses.

## Abstract 2

Protein-protein interactions (PPIs) in the NMDAR complexes are mechanistic substrates for interactions and convergence of synaptic risk genes in schizophrenia. Here, we present the first example of leveraging altered PPIs in schizophrenia by targeting Src-PSD-95 association and enhancing molecular, electrophysiological and behavioral phenotypes of NMDAR function.

## Introduction

Hypofunction of N-methyl-D-aspartate receptor (NMDAR) signaling has been implicated for many neuropsychiatric illnesses such as intellectual disability, age-related cognitive decline, and schizophrenia (1). Various strategies and candidate agents have, thus, been investigated to enhance NMDAR function (2), although agents that are efficacious for specific diseases are yet to be identified. Previously tested agents for intervention in NMDAR hypofunction are endogenous coagonists and positive allosteric modulators (PAMs) of the receptors. These agents activate NMDARs regardless of cellular context, and they trigger diverse signaling pathways and cellular functions depending on the location of the receptors, i.e., subcellular locale or on specific cell types (3, 4). Consequently, these agents are limited in their ability to target disease-specific mechanisms and to prevent unwanted outcomes.

A more focused, context-specific strategy is to target molecular events that are upstream of the receptor and dysregulated in diseases. Specifically, protein-protein interactions (PPIs), which determine the proximity and association between molecules, are a promising target as they modulate the interactions between signaling molecules that are essential regulators of their functions (2, 5). In addition, altered PPIs can reflect or harbor disease processes of complex neuropsychiatric illnesses, in which genetic variants cause illnesses in many different combinations (6–8). Therefore, pathophysiologic processes arise not only from the risk genes but also from their aberrant interactions which are often manifested by their altered PPIs.

For instance, schizophrenia is caused by hundreds of risk genes, many of which are shared by other neuropsychiatric illnesses (9, 10), and yet these risk genes precipitate the illness via specific modes of interactions and convergence (6, 7, 11). Presently, mechanistic substrates for their interactions and convergence are unknown and have not been exploited as an avenue for new therapeutic strategies for schizophrenia. In the present study, we present a proof-of-concept study in which perturbation of PPIs in the receptor complexes rescues NMDAR hypofunction phenotypes.

Our prior studies demonstrated that altered protein interactions in NMDAR complexes converge and reduce the activity of the nonreceptor tyrosine kinase, Src, and precipitate NMDAR hypofunction in schizophrenia (12). We found that the dorsolateral prefrontal cortex (DLPFC) of individuals with schizophrenia exhibit a striking decrease in tyrosine phosphorylation of the GluN2 subunits, which is indicative of decreased NMDAR function. This functional dysregulation of NMDARs was found to be mediated by hypoactivity of Src. This hypoactivity in turn was not associated with decreased expression of Src or its interactors but accompanied by increased association of Src with PSD-95 and erbB4 and decreased Src association with rPTPα GluN2A and dysbindin1 (12). Each of these aberrant protein interactions can reduce Src activity (12) and subsequently affect NMDAR currents through phosphorylation of NR2 subunits.

Here, we ask if these altered protein associations could be leveraged to rescue NMDAR hypofunction in schizophrenia. Of the protein interactions described above, the Src–PSD-95 interaction is of particular interest as a potential therapeutic target since PSD-95 is increased in NMDAR complexes in schizophrenia and PSD-95 inhibits Src activity through direct interaction with the SH2 domain of Src (13). We designed and tested a strategy to selectively target and reduce the interaction between Src and PSD-95 and, thereby, to selectively enhance Src activity in the NMDAR complexes. To enhance Src activity, we employed a Src activating PSD-95 inhibitory peptide (SAPIP) comprising the Src SH2 domain with a R175K mutation to minimize phosphotyrosine binding (14). SAPIP prevents PSD-95 from binding to Src, thereby overcoming inhibition of Src activity (14). This study tests the hypotheses that an interfering peptide intervention in the PSD-95–Src association will (a) normalize ex vivo synaptic currents, (b) improve cognitive behavioral and electrophysiological phenotypes in vivo in mice, and (c) enhance Src activity in postmortem synaptoneurosomes of human prefrontal cortex.

## Results

### TAT-SAPIP enhances Src activity by blocking Src binding to PSD-95.

**Figure 1:**
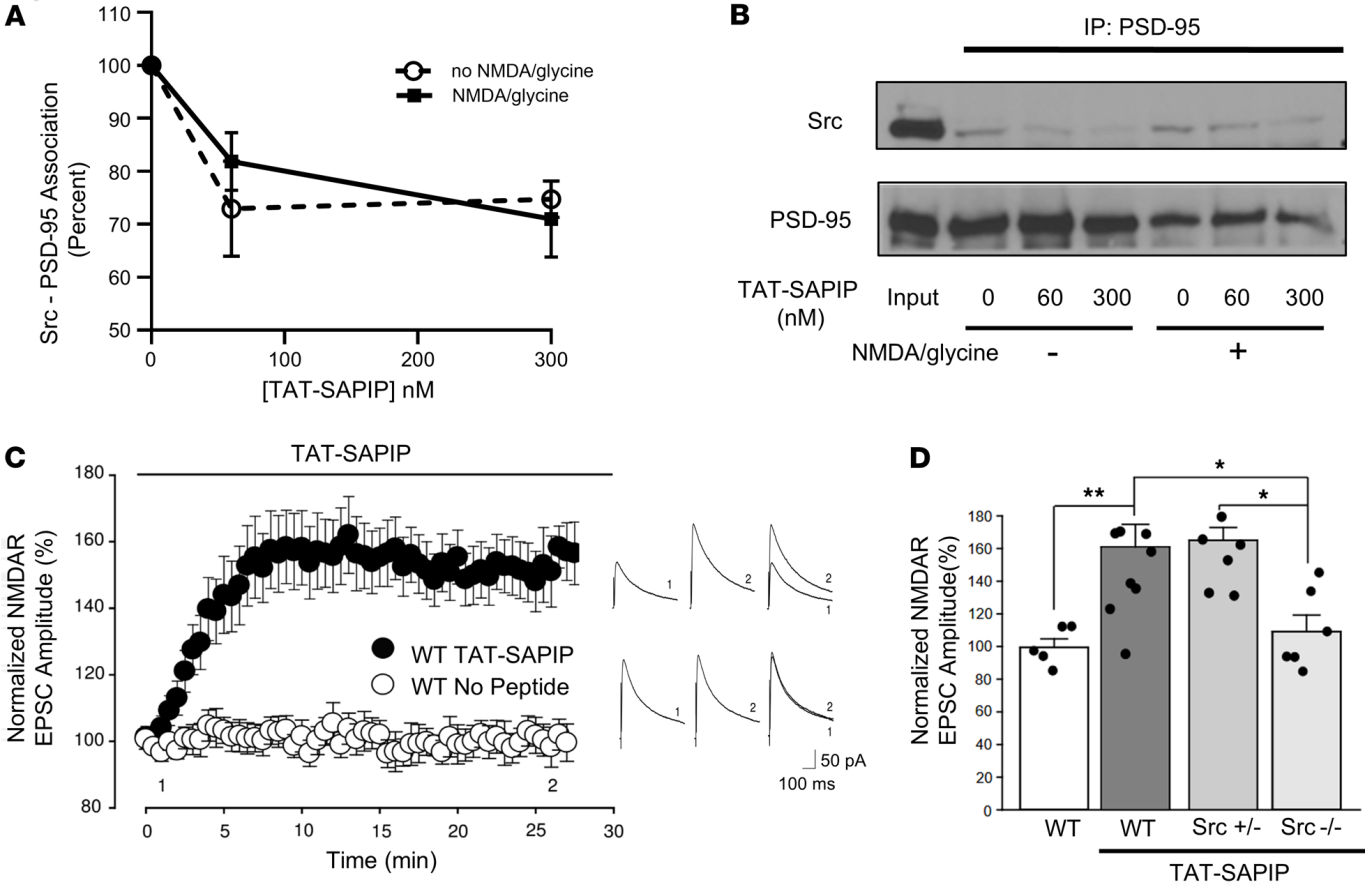
TAT-SAPIP reduces Src–PSD-95 association, and TAT-SAPIP rescues decreased synaptic NMDAR currents. (**A** and **B**) The effects of TAT-SAPIP on Src–PSD-95 association. Rat primary cortical neurons were treated with TAT-SAPIP with or without 10 μM NMDA + 1 μM glycine. Synaptosomal extracts were immunoprecipitated for PSD-95 and probed for Src. Src–PSD-95 association was significantly decreased in the TAT-SAPIP treatment group. (**A**) Graphic representation. (**B**) Representative blots. (**C**) Scatter plot of NMDAR EPSC peak amplitude over time from WT mice CA1 pyramidal neurons with or without intracellularly applied TAT-SAPIP peptide. Black bar above the plot indicates the duration of intracellular peptide application. Representative average NMDAR EPSC traces recorded at membrane potential of +60 mV at the times indicated (1 and 2). (**D**) Histogram representation of normalized NMDAR EPSC amplitude for each group at times denoted by the number 2 in **C**. Significant difference was observed in WT treated with TAT-SAPIP (161.1% ± 13.8% of baseline, *n* = 10) versus without TAT-SAPIP (99.4% ± 5.2% of baseline, *n* = 5 without peptide) (*P* = 0.008). With TAT-SAPIP treatment, significance was detected between WT (161.1% ± 13.8% of baseline, *n* = 10) and *Src^–/–^* (109.2% ± 10.0% of baseline, *n* = 6, *P* = 0.013). Significant difference was also observed between *Src^+/–^* (165.0% ± 9.4% of the baseline, *n* = 8) and *Src^–/–^* groups both treated with TAT-SAPIP (*P* = 0.014). No differences were observed between WT and *Src^+/–^* when treated with TAT-SAPIP. Black dots represent individual data points. *P < 0.05, **P < 0.01. One-way ANOVA followed by Holm-Šidák post hoc test was used for statistic comparisons in **D**. Data represent mean ± SEM.

To intervene the Src–PSD-95 association in the synapse, we devised a cell-penetrating form of SAPIP. We fused the membrane-transduction sequence of HIV-TAT to the N-terminus of SAPIP (TAT-SAPIP). We first examined the ability of TAT-SAPIP to reduce Src–PSD-95 association when applied extracellularly. Primary rat cortical neurons were treated with TAT-SAPIP in the presence or absence of NMDA (10 μM) and glycine (1 μM). Synaptoneurosomes from these cells were analyzed for protein associations between Src and PSD-95 using immunoprecipitation. We found significantly decreased Src–PSD-95 association at 60 nM and 300 nM in the absence of NMDA receptor activation [F(2,12) = 7.46, *P* = 0.012, 0.019 respectively], as well as in the presence of it [F(2,12) = 7.919, *P* = 0.072, 0.005, respectively] (Figure 1, A and B). Thus, extracellular application of TAT-SAPIP decreases Src–PSD-95 association in neurons.

### TAT-SAPIP increases synaptic NMDAR activity.

TAT-SAPIP could increase synaptic NMDAR activity because TAT-SAPIP derepresses Src, which may in turn increase NMDAR activity. Src increases NMDAR activity in cultured neurons (15) and ex vivo slices of the hippocampus (16, 17) and the medial PFC (Supplemental Figure 1; supplemental material available online with this article; https://doi.org/10.1172/jci.insight.189634DS1). To test the effects of TAT-SAPIP on NMDAR activity directly, we administered TAT-SAPIP in hippocampal slices while recording postsynaptic responses at the Schaeffer collateral–CA1 synapses (16). Importantly, we found that TAT-SAPIP had no effect per se on the efficacy of synaptic transmission. Excitatory postsynaptic potentials (EPSPs), which are mediated by AMPA receptors, were unaffected by bath applying TAT-SAPIP as assessed in either field or whole-cell patch recordings (Supplemental Figure 2). To determine the effects of TAT-SAPIP on NMDAR-mediated synaptic responses, we measured pharmacologically isolated NMDAR excitatory postsynaptic currents (EPSCs). TAT-SAPIP was applied directly through the patch pipette during whole-cell voltage-clamp recordings (Figure 1C). We found that NMDAR EPSCs gradually increased to 161% ± 14% of the basal level (*P* = 0.008, TAT-SAPIP versus no peptide; *P* = 0.002, TAT-SAPIP versus baseline) and remained stable for the duration of the recording (Figure 1C).

### TAT-SAPIP is ineffective in Src^–/–^ mice.

To confirm that this increase in NMDAR EPSCs is dependent on Src, TAT-SAPIP was tested in hippocampal slices from homozygous Src-KO (*Src*^–/–^) mice (Figure 1D). In contrast to its effect in WT neurons, TAT-SAPIP failed to affect NMDAR EPSCs in *Src*^–/–^ neurons (109% ± 10% of baseline, *P* = 0.4, TAT-SAPIP versus baseline; *P* < 0.05, *Src*^+/–^ versus WT). Thus, TAT-SAPIP enhancement of NMDAR currents is dependent on the presence of *Src* (15). We have previously shown that heterozygous Src (*Src*^+/–^) mice exhibit a subset of the behavioral deficits observed in schizophrenia (18). Therefore, we assessed whether TAT-SAPIP can affect synaptic NMDAR activity in these mice. We found that TAT-SAPIP increased NMDAR EPSC amplitude to 165% ± 9.4% from baseline in *Src^+/–^* neurons (*P* = 0.014, *Src*^+/–^ versus *Src*^–/–^) (Figure 1D). Thus, TAT-SAPIP enhances NMDAR currents in neurons with only a single copy of *Src,* which was used to create a model of decreased Src activity.

### TAT-SAPIP rescues NMDAR hypoactivity in Sdy^–/–^mice.

**Figure 2:**
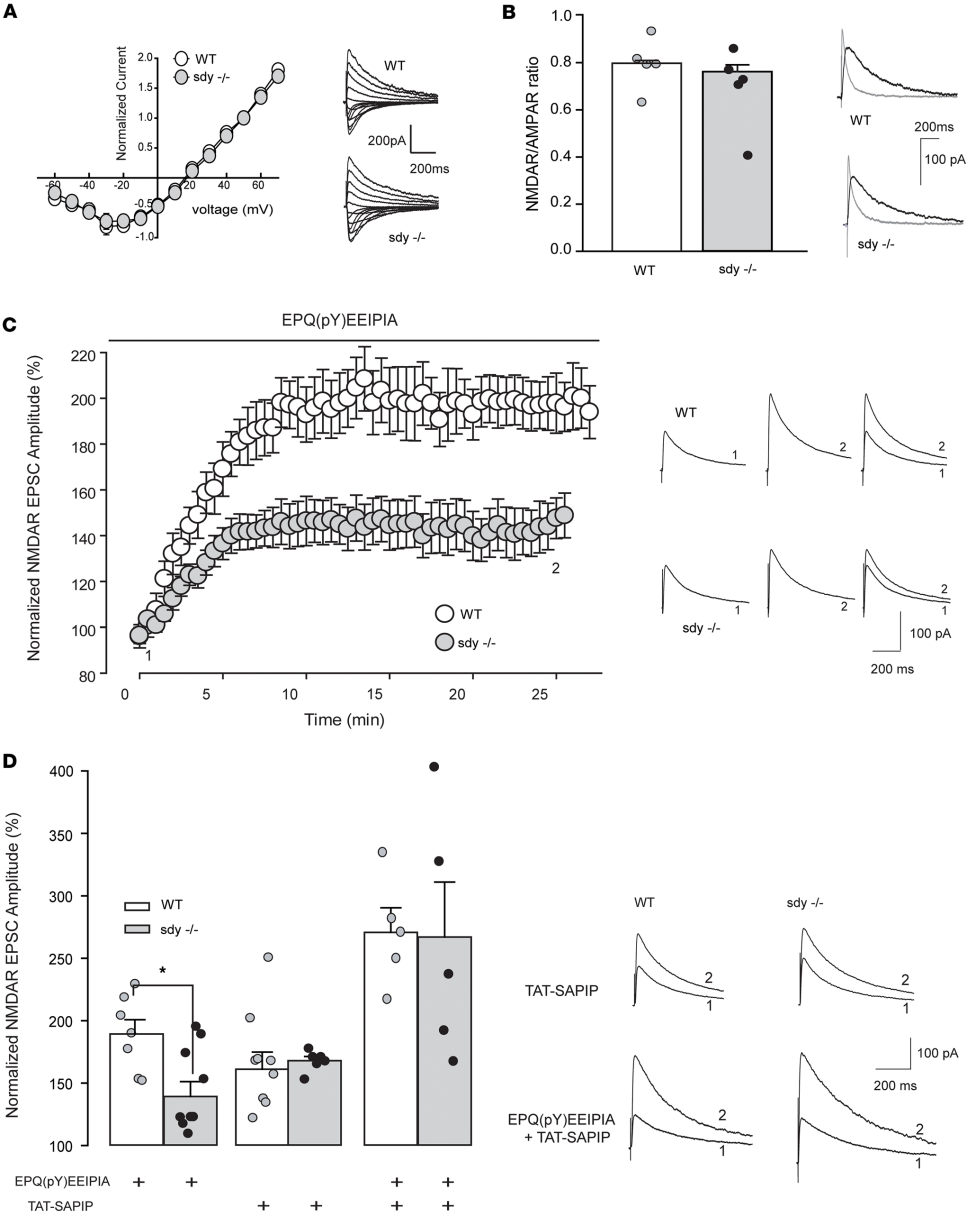
SAPIP rescues NMDAR hypofunction in *Sdy^–/–^* mice. (**A**) Scatter plot with representative traces showing the current-voltage (I- V) relationship and reversal potential of NMDAR EPSCs at Schaeffer collateral-CA1 synapses from *Sdy^–/–^* and WT mice. (**B**) NMDAR versus AMPAR current are not different between WT and *Sdy^–/–^* mice. Representative traces of AMPAR current (gray) and NMDAR current (black) from each genotype are displayed above. Black dots represent individual data points. (**C**) Scatter plot of NMDAR EPSC peak amplitude recorded with intracellularly applied EPQ(pY)EEIPIA peptide from dysbindin knockout (gray) and WT mice (white). Black bar indicates the duration of peptide application in both genotypes. Representative average NMDAR EPSC traces were recorded at membrane potential of +60 mV at the times indicated (1 and 2). (**D**) Histogram representation of normalized NMDAR EPSC amplitude over time recorded from CA1 pyramidal neurons taken from dysbindin knockout (gray) and WT mice (white) with intracellularly applied EPQ(pY)EEIPIA peptide alone (WT 196.6% ± 12.2% of baseline, *n* = 7; *Sdy^–/–^* 149.6% ± 9.3 % of baseline, *n* = 11; *P* = 0.01, Student’s *t* test), TAT-SAPIP peptide alone (WT 161.1% ± 13.8% of baseline, *n* = 10; *Sdy^–/–^* 167.9% ± 3.7% of baseline, *n* = 7, Student’s *t* test) or with both EPQ(pY)EEIPIA + TAT-SAPIP peptides (WT 270.8% ± 22.0% of baseline, *n* = 5; *Sdy^–/–^* 267.0% ± 39.4% of baseline, *n* = 5, Student’s *t* test). Representative average NMDAR EPSC traces from TAT-SAPIP and EPQ(pY)EEIPIA + TAT-SAPIP–treated groups recorded at membrane potential of +60 mV at baseline (labeled 1) versus 25 min (labeled 2). All measurements were taken from distinct samples. Black dots represent individual data points. Data represent mean ± SEM.

Next, we tested whether TAT-SAPIP can enhance NMDA receptor function in other conditions that are related to schizophrenia. Dysbindin-1 is decreased in the DLPFC (12) of schizophrenia (19), and mice lacking dysbindin-1, *Sdy*^–/–^ mice, have multiple neurobiological deficits implicated for schizophrenia, including NMDA receptor hypofunction (12, 20, 21) and decreased Src activity (12). Like *Src^–/–^* mice, *Sdy^–/–^* mice did not exhibit changes in NMDAR current-voltage characteristics nor did they show any changes in NMDAR/AMPAR ratios (Figure 2, A and B). Src-mediated increases in NMDAR activity can be induced by delivering a Src kinase activating peptide, EPQ(pY)EEIPIA, intracellularly (15). EPQ(pY)EEIPIA induced enhancement of NMDAR EPSC amplitude was much reduced in *Sdy^–/–^* mice compared with WT (149.6% ± 9.3% versus 196.6% ± 12.2%, respectively) (Figure 2C). This may suggest a decrease in the activatable pool of Src at excitatory synapses in *Sdy^–/–^* mice. In the presence of TAT-SAPIP, however, NMDAR EPSC amplitude in *Sdy^–/–^* mice was increased to a level indistinguishable from that of WT (167.9% ± 3.7% versus 161.1% ± 13.8%, respectively) (Figure 2D). In addition, coadministration of EPQ(pY)EEIPIA and TAT-SAPIP resulted in comparable potentiation of NMDAR EPSC amplitudes in neurons from both *Sdy^–/–^* and WT controls (267.0% ± 39.4% versus 270.8% ± 22.0%, respectively; Figure 2D). These results suggest that TAT-SAPIP rescues Src hypoactivity of NMDARs in mouse models that may recapitulate pathophysiologic mechanisms of schizophrenia.

### TAT-SAPIP rescues ERP and cognitive deficits in Src^+/–^ mice.

**Figure 3:**
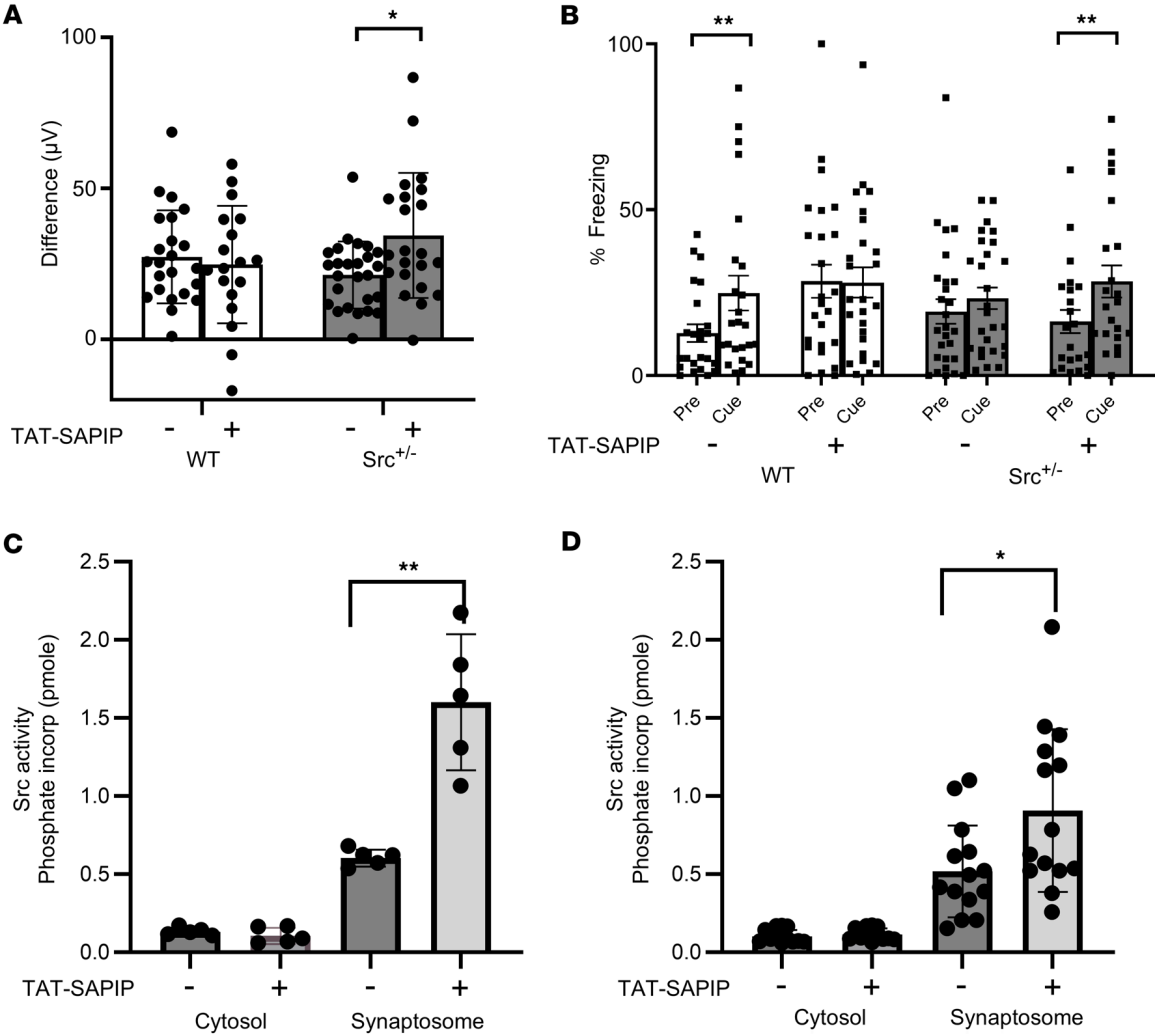
TAT-SAPIP rescues deficits in auditory event–related potentials and TFC in Src^+/–^ mice and enhances Src activity in the DLPFC of patients. (A) Event-related potential response to a novel versus a repeated (standard) stimulus. Response to the novel stimulus was increased in Src+/– mice following TAT-SAPIP. *P < 0.05. Group sizes were 22 WT vehicle (11 male, 11 female), 26 Src^+/–^ vehicle (14 male, 12 female), 23 WT SAPIP (12 male, 11 female), and 22 Src^+/–^ SAPIP (12 male,10 female). No effect was observed for sex. Data were analyzed using a 3-way ANOVA with genotype, sex, and treatment as variables. (B) TFC in WT and Src^+/–^ mice following vehicle or TAT-SAPIP. WT mice treated with vehicle showed significantly more freezing during the cue relative to precue period (**P < 0.01), which was not seen in Src ^+/-^ mice. TAT-SAPIP restored TFC in Src^+/–^ mice (**P < 0.01). Group sizes were 24 WT vehicle( 11 male, 13 female), 27 Src^+/–^ vehicle (12 male, 15 female), 25 WT SAPIP (14 male, 11 female), and 21 Src^+/–^ SAPIP (10 male, 11 female). No effect was observed for sex. Data were analyzed 3-way repeated measures ANOVA. (C and D) The effects of TAT-SAPIP on Src activity. Synapto-neurosomes or cytosol from the PFC of mice (C) or from the DLPFC of 14 humans (D) were incubated with 200 ng of TAT-SAPIP and Src activity was measured as previously described (12). SAPIP significantly increased Src activity in synaptoneurosomes derived from mouse (266.6% ± 32.2% of base line, *n* = 10, *P* = 0.0009) or human PFC (170.0% ± 29.4%, *n* = 30, *P* = 0.024). Bar graphs in C and D show mean and standard error of the mean. For A–D, dots represent individual data points and data represent mean ± SEM.

We then tested if TAT-SAPIP can rescue behavioral phenotypes associated with Src hypoactivity, particularly those associated with schizophrenia. Deficits in the P3a auditory event-related potential (ERP) difference waveform are associated with impaired cognitive performance and have been demonstrated in schizophrenia (22). To test the potential effect of TAT-SAPIP, we administered this peptide intracerebroventricularly (ICV) for 14 days (Figure 3A). We found that TAT-SAPIP did not alter P3a amplitude difference in WT mice. However, TAT-SAPIP significantly increased P3a amplitude difference in *Src*^+/–^ mice (Figure 3A). These data indicate that TAT-SAPIP enhances the neural circuitry underlying P3a in Src-deficient but not WT mice. P3a is a well-established probe for sensory processing abnormalities in schizophrenia (22). TAT-SAPIP appears to have restorative effects on the P3a deficits in *Src^+/–^* mice, while disrupting normal function in WT mice (Figure 3A) (gene × treat [F(1,82) = 5.51, *P* = 0.021]). No differences were observed for sex.

Schizophrenia is characterized by deficits across several cognitive domains subserved by the hippocampus and prefrontal cortex. Trace fear conditioning (TFC) is a hippocampus-PFC dependent cognitive processes in which the presentation of a cue (conditioned stimuli [CS]) and a shock (unconditioned stimuli [US]) are separated by a stimulus-free interval (trace period) (23, 24). Successful association of the cue and shock requires retention of the cue across the trace period. A repeated-measures ANOVA using percent time freezing during precue versus cue periods found a significant 3-way interaction between gene, treatment, and cue [F(1,89) = 6.58, P = 0.012]. No significant effects were observed for sex. WT mice treated with vehicle showed a significant increase in freezing during the cue compared with precue period (*P* = 0.003) (Figure 3B). In contrast, Src^+/–^ mice failed to show such differences between the cue and precue (*P* = 0.287), exhibiting a deficit in associating CS and US over a trace period. Chronic ICV TAT-SAPIP restored TFC in Src^+/–^ mice, as indicated by significantly increased freezing to the cue versus precue (*P* = 0.004). However, TAT-SAPIP disrupted TFC in WT mice, which showed high freezing during both cue and precue periods (*P* = 0.92) (Figure 3B). Thus, TAT-SAPIP rescued cognitive behavioral deficits associated with Src hypofunction, while disrupting function in WT mice. This finding suggests that there is a classical inverted U-shaped response profile with an optimal state that is disrupted by either increased or decreased NMDAR function. Additionally, we assessed whether mice in each condition showed significantly greater freezing during the cue versus precue period by calculating the difference between percent freezing during the cue minus the precue period. This was then assessed in a series of 1-sample *t* tests to determine whether the resulting difference was significantly greater than zero. Both WT vehicle and Src^+/–^ peptide mice showed difference values significantly greater than zero (*P* = 0.008 and 0.0062, respectively). Neither the Src^+/–^ vehicle or the WT peptide showed significance (*P* = 0.32 and 0.917, respectively). This suggests that the mice in both the WT vehicle and Src^+/–^ peptide groups showed more freezing during the cue than the precue period.

### TAT-SAPIP enhances Src activity in postmortem brains of individuals with schizophrenia.

We examined if TAT-SAPIP can enhance Src activity in brains of individuals with schizophrenia. We have previously established a protocol to examine synaptoneurosomes from postmortem brains to monitor their responses to signaling activation (25). We first tested frozen mouse brain tissues. We tested the effects of TAT-SAPIP in the synaptoneurosomes where Src associates with PSD-95, compared with the cytosol where Src does not associate with PSD-95 (26). We treated the synaptoneurosomes or cytosol derived from mouse prefrontal cortex with TAT-SAPIP or vehicle and assessed Src activity. Src activity differed significantly between the 4 groups = F(3,16) = 49.95, *P* < 0.001]. TAT-SAPIP increased Src activity in the synaptoneurosomes [t(8) = 5.088, *P* = 0.0009], while it did not in the cytosol [t(8) = 0.9172, *P* = 0.98; Figure 3C).

We then examined the synaptoneurosome and cytosol derived from the postmortem DLPFC of 14 patients (7 with schizophrenia and 7 age- and sex-matched controls) (Figure 3D). In all 14 patients together, Src activity differed significantly between the 4 groups (cytosol, synaptoneurosomes each in the presence and absence of TAT-SAPIP [F(3,50) = 19.20, *P* < 0.001]). We then examined if TAT-SAPIP could increase Src activity differentially between individuals with schizophrenia and controls. We conducted 2-way repeated-measures ANOVA in which the condition (control versus schizophrenia) was a between-patient factor and treatment (vehicle versus SAPIP) was a within-patient measure (Supplemental Figure 3). A significant effect was observed for treatment, with SAPIP significantly increasing Src activity [F(1,12) = 13.7, *P* = 0.003). No significance was seen for condition or for the interaction between treatment and condition. Pairwise contrasts between the 4 cells showed significant increases induced by SAPIP in both control and schizophrenia groups but no significant difference between control versus schizophrenia either under vehicle or SAPIP (Supplemental Figure 4).

## Discussion

Receptor modulation using cofactors and ligands has been the main therapeutic strategy not only for diseases associated with NMDAR dysfunction but also for most other neuropsychiatric illnesses. These include agonists or antagonists for serotonergic receptors (5-HT1A, 5-HT2A, 5-HT2C) (27–29), muscarinic receptors (M1, M2/3, M4,5) (30, 31), nicotinic receptors (a7) (32, 33), and glycine site of NMDA receptors (D-serine) (3, 4, 34). Increasing genetic evidence, however, indicates that common neuropsychiatric illnesses, such as schizophrenia, are not caused by a single gene or neurotransmitter system but results from the interactions and convergence of many risk genes, impacting fundamental biological processes (35, 36), such as synaptic activity and structure (6–8, 37). This calls for a shift in paradigm in the design of pharmacologic strategies from targeting individual genes or receptors to modifying the interactive dynamics between risk genes.

Targeting of PPIs in the synapse is based on their crucial roles in synaptic activity/plasticity as well as on their alterations observed in schizophrenia (38–40). PPIs, particularly in the PSD, are mechanistic substrates for numerous molecular interactions underlying synaptic function (41, 42). Therefore, small changes in risk gene products in the PSD can alter PPIs in key signaling networks/interactomes. Indeed, NMDA receptor complexes captured by GluN1–immunoprecipitation from the DLPFC showed increases in PSD-95 and erbB4 while it showed decreases in GluN2A and rPTPα, each of which can decrease Src activity via their PPIs. Of these, we chose to target Src–PSD-95 association because it mediates Src inhibitory effects of PSD-95.

Src–PSD-95 association occurs in macromolecular complexes in the PSD, where many proteins are tightly linked to each other. It is a challenge to introduce peptides into the PSD microdomain and perturb protein associations therein. TAT is a cationic peptide that interacts with negatively charged components in the cellular membranes and transports cargo molecules into the cell. We found that extracellular application of TAT-SAPIP reduces Src–PSD-95 association in rat cortical neurons (Figure 1, A and B). Thus, our results demonstrate that PPIs in macromolecular complexes in the PSD can be modified by peptides fused with cell-penetrating peptides.

TAT-SAPIP decreased Src–PSD-95 association both in the presence and absence of NMDA + glycine (Figure 1, A and B). This may suggest that concurrent activation of NMDAR does not drastically affect the effects of TAT-SAPIP. Given that the cells were treated with TAT-SAPIP by bath application for 45 minutes, it is possible that our assay did not capture the acute effect of NMDA receptor activation on modulation of Src–PSD-95 association by TAT-SAPIP. Interestingly, NMDA receptor activation appears to decrease the Src–PSD-95 complexes in the synaptoneurosome (Figure 1B). This is likely because of the internalization of NMDAR complexes in response to sustained activation of the receptors in this experiment as a result of homeostatic plasticity (43, 44).

TAT-SAPIP enhances NMDAR currents but not AMPA currents (Figure 1C) and such effects are shown only in the presence of Src (Figure 1D). Thus, TAT-SAPIP enhances NMDAR currents specifically mediated by Src. To test these effects of TAT-SAPIP beyond the condition of reduced Src expression, we examined mice perturbed for Dysbindin-1, *Sdy^–/–^*, which have multiple neurobiological deficits implicated for schizophrenia and altered NMDA receptor signaling (12, 19). TAT-SAPIP indeed enhances NMDAR currents in *Sdy^–/–^* mice. Interestingly, this is via increasing the activatable pool of Src (Figure 2) and, therefore, it can be effective in instances of schizophrenia in which NMDAR activity is attenuated by mechanisms that do not affect Src directly in schizophrenia.

Our results suggest that such enhancement of NMDAR currents could modify sensory processing abnormalities in schizophrenia (Figure 3). TFC includes a trace interval between tone (conditioned stimulus [CS]) offset and shock (unconditioned stimulus [UCS]). Retention of the cue during the trace interval is dependent on working memory, since the strength of TFC learning decays as the length of the trace interval increases (44–46). Acquisition of TFC is critically dependent upon NMDARs (47, 48), both GluN2A and GluN2B (49). It is likely that Src reduction disrupts TFC via decreasing activity at GluN2 containing receptors as shown in Src hypomorphs (Figure 3). Conversely, TAT-SAPIP restores TFC by enhancing the ability of Src to activate GluN2-containing receptors (Figure 3). Unexpectedly, TAT-SAPIP disrupted TFC in WT mice. This finding may suggest that there is an inverted U-shaped response profile with an optimal state that is disrupted by either increased or decreased Src activity. TAT-SAPIP–mediated rescue of deficient TFC raises the possibility of an important role for the Src–PSD-95 association in memory and cognitive dysfunction implicated in schizophrenia.

We asked if Src–PSD-95 association could be leveraged to enhance Src activity in individuals with schizophrenia. Given that Src kinase is involved in oncogenesis (50, 51), the effects of TAT-SAPIP on Src kinase should be specific to the synapse. As a first step, we tested this in the synaptoneurosomes versus cytosol derived from the DLPFC of individuals with schizophrenia and controls. As shown in mice (Figure 3C), TAT-SAPIP fails to enhance Src activity in the cytosol of DLPFC, where Src is not associated with PSD-95 and, thus, cannot be affected by TAT-SAPIP (Supplemental Figure 4). In contrast, TAT-SAPIP significantly increased Src activity in the synaptoneurosomes from 14 patients, 7 individuals with schizophrenia and their matched controls (*P* = 0.024). Thus, the perturbation of the Src–PSD-95 association can enhance Src activity specific to the synapse. These effects of TAT-SAPIP in human PFC are further supported by our finding that NMDAR EPSCs in neurons in the medial PFC are dynamically regulated by Src kinase activity: activating Src increases NMDAR EPSCs and suppressing Src activity reduces NMDAR EPSCs (Supplemental Figure 1).

We then asked if TAT-SAPIP could increase Src activity differentially between schizophrenia and controls. A repeated-measures ANOVA indicated that SAPIP does increase Src activity in both controls and individuals with schizophrenia, yet there was no differential effect of SAPIP between the 2 groups. This is consistent with our findings in mice in which TAT-SAPIP enhances NMDAR currents in WT as well as in *Src^+/–^* (Figure 1, C and D) and modulates behaviors in mice of both genotypes (Figure 3B).

There are several limitations that define the scope of data interpretation. Results from human tissues are based on examination of a small sample size. In addition, our findings on the role of Src activity and TAT-SAPIP are only from 2 brain regions. While TFC involves the hippocampal PFC circuit, our human studies are focused on and limited to the PFC. Electrophysiological studies demonstrate the role of Src activity and its PPIs on NMDAR function in the PFC as well as in the hippocampus. The effect of TAT-SAPIP on NMDAR activity shown in the hippocampus is likely to be observed in the PFC. Whether this will be the case in other brain regions is also left for future studies.

This study was designed to test the perturbation of PPIs altered in a neuropsychiatric illness as a new strategy to modify disease phenotypes. Our results demonstrate that inhibiting the Src–PSD-95 association rescues molecular, electrophysiological, and behavioral phenotypes of NMDAR signaling deficits relevant to schizophrenia. While there are limitations to TAT-SAPIP as a potential therapeutic agent, we here show proof of concept that specific PPIs in the synapse can be leveraged to modify behavioral phenotypes. We thus propose that PPI subnetworks of disease relevant pathways — particularly in the synapse, where risk genes of various illnesses converge — be considered as therapeutic targets.

## Methods

### Sex as a biological variable.

in vivo experiments used both female and male mice. All statistical analyses were conducted using sex as an independent variable. Human postmortem tissue was derived from both sexes for patients and healthy controls. Analysis of this data for sex is not feasible due to the relatively small sample size. In vitro slice studies were done in both female and male mice. Mouse coimmunoprecipitation and Src activity studies were only conducted in male mice. It is expected that the results of these studies will be relevant to both female and male patients.

### Study design.

The goal of the current studies was to assess the effectiveness of using TAT-SAPIP to target a PPI thought to regulate NMDAR function. It was predicted that TAT-SAPIP would penetrate the cell membrane and increase Src activity. It was predicted that TAT-SAPIP would enhance Src activity in synaptoneurosomes derived from mouse cortex and postmortem DLPFC tissues of patients. It was predicted that the effectiveness of TAT-SAPIP would depend directly on Src activity and, therefore, TAT-SAPIP would be ineffective in *Src^–/–^* mice. Furthermore, it was predicted that this would lead to increases in NMDAR activity in hippocampal slice tissue. Finally, it was predicted that TAT-SAPIP would rescue ERP and cognitive deficits seen in *Src^+/–^* mice.

Sample sizes are reported in figure captions and were determined based on historical precedence to be the minimal size to detect likely meaningful differences in similar experiments. For P3a and TFC, all mice born during the period of the experiment were used. Both experiments were stopped prior to data analysis. No data were excluded from IP, Western blot, or Src activity assays. All Src activity assays were conducted in technical duplicates. No data were excluded from TFC. For EEG experiments, mice were removed if the headcap became detached from the skull or if the EEG recording failed to show an expected ERP signal using preestablished criteria developed in the Siegel lab. EEG recordings were assessed for quality by 3 individuals. For P3a and TFC, a Grubbs outlier test was run. One mouse was removed from the P3a experiment due to an excessively high P3a waveform. No mice were removed from the TFC experiment.

### Postmortem brains.

Flash-frozen DLPFC tissues from 14 patients (7 individuals with schizophrenia, and 7 controls), obtained from the Penn Brain Bank at the University of Pennsylvania were used for the study. Approved by the IRB at the University of Pennsylvania, patients were prospectively diagnosed by Diagnostic and Statistical Manual of Mental Disorders, 4th Edition (DSM-IV), and consents for autopsy were obtained from the next of kin or a legal guardian. Patients with a history of substance abuse, neurological illnesses, or the need for ventilator support near death were excluded. Detailed demographic data are shown in Supplemental Table 1.

### Animals.

Src heterozygous C57BL/6 mice were obtained from The Jackson Laboratory (strain no. 002277) and bred to acquire genotypes *Src^–/–^*, *Src^+/−^* and WT (*Src^+/+^*) (53). Mice with a homozygous KO of DTNBP1 (*Sdy*^–/–^), which produces Dysbindin-1, were obtained from a colony derived from mice originally obtained from The Jackson Laboratory. Mice were housed in ventilated cages on a 12:12-h light/dark cycle, with lights on from 7 a.m. to 7 p.m. All ex vivo recordings and in vivo testing occurred during the light cycle. All procedures related to ex vivo recordings were performed at the Hospital for Sick Children in accordance the Canadian Council on Animal Care and approved by the Animal Care Committee of the Hospital for Sick Children.

### Rat cortical primary culture.

Primary cultures of rat cortical neurons were obtained from the Mahoney Institute of Neurological Sciences Neuron Culture Service at the Perelman School of Medicine. The neurons were plated at a density of 350,000 cells/mL in Neurobasal media supplemented with B27.

### Preparation of SAPIP.

The SAPIP was generated as GST-fusion proteins as described previously(14) or as TAT-SAPIP with Tat protein transduction domain (YGRKKRRQRRR) attached to the N-terminal of the SAPIP. GST fusion proteins were expressed in *E. coli* and purified by GST affinity chromatography (GE) as previously described by having them expressed in bacteria and according to the manufacturer’s instructions. TAT-SAPIP was purified by Nickel affinity chromatography (Qiagen) following the protocols from the manufacturer.

### SAPIP induced activation of Src.

Cultured rat cortical neurons were washed with 1x PBS and treated first with 0, 60, and 300 nM of TAT-SAPIP in Krebs Ringer (KR) solution at 37°C for 15 minutes. This was followed by further incubation in the presence or absence of 10 μM NMDA and 1 μM Glycine at 37°C for 30 minutes. The cells were collected and processed to obtain synaptosomal fractions for immunoprecipitation and western blotting as described below.

In mouse or human postmortem brains, synaptoneurosomes and cytosols were obtained from the PFC of C57BL/6 mice or postmortem DLPFC from 14 patients. Twenty milligrams of synaptoneurosomes or cytosol were incubated in KR solution with 200 ng of GST-SAPIP or GST at 4°C overnight.

### Src activity assay.

Following the incubation with or without SAPIP, synaptoneurosomes from mouse or human postmortem brains were assayed for Src activity according to the manufacturer’s instructions for the Src assay kit (Upstate) (12). The samples were added with the Src kinase substrate peptide (150 μM, final concentration, Src kinase reaction buffer and [γ-^32^P]ATP; Amersham, 30 picomol, 3000 Ci/mmol) and incubated at 30°C with agitation. Src kinase activity was evaluated by measuring incorporation of [γ-^32^P]ATP into the substrate by TCA precipitation and scintillation counting.

### Subcellular fractionation, Western blotting, and immunoprecipitation.

Synaptoneurosome, synaptic membranes, or PSD enriched fractions were isolated from the prefrontal cortex of the mouse or rat cortical cultures by methods from previous studies (12, 26). Soluble and insoluble fractions were collected and analyzed by immunoblotting. Synaptic membrane (P2) enriched fractions were immunoprecipitated with anti–PSD-95 (antibodiesinc; 75-028) and mixed with agarose-conjugated protein A-G beads. Solubilized immunoprecipitates were size separated and immunoblotted with anti–PSD-95 (antibodiesinc; 75-028). Western Blotting was also carried out as indicated in the figures for GluN1/GluN2A (Santa Cruz, sc-518053, sc-390094) or Src (Cell Signaling, 2110s). Signals were detected with ECL (Amersham), developed on x-ray film, and quantified using densitometric scanning.

### Hippocampal and PFC ex vivo slice electrophysiology.

Parasagittal hippocampal slices (300 μm) or coronal cortical slices (320 μm) were prepared in ice-cold ACSF from mice (22–28 days of age) anesthetized (20% [wt/vol] urethane, i.p.). Slices were placed in a holding chamber (30°C) for 40 minutes and then allowed to passively cool down to room temperature (21°C–22°C for ≥ 30 min) before recording. A single slice was transferred to a recording chamber and superfused with ACSF at 4 mL min^–1^ composed of (in mM) 124 NaCl, 2.5 KCl, 1.25 NaH_2_PO_4_, 2 MgCl_2_, 11 D-glucose, 26 NaHCO_3_, and 2 CaCl_2_ (ACSF chemicals from Sigma-Aldrich) saturated with 95% O_2_ (balance 5% CO_2_) at room temperature, pH 7.40, osmolality 305 mOsm. Whole-cell patch-clamp recordings of CA1 pyramidal neurons, or from layer 5 medial PFC pyramidal neurons, were carried out using the visualized method (Zeiss Axioskop 2FS microscope). Patch pipettes (4–5 MΩ) solution containing (in mM): Cs gluconate 117, CsCl 10, BAPTA 10, CaCl_2_ 1, HEPES 10, ATP-Mg 2, QX-314 10, and GTP 0.3 (pH 7.25, osmolality 290 mOsm). Synaptic responses were evoked in hippocampus by stimulating Schaffer collateral afferents using bipolar tungsten electrodes located ~50 μm from the pyramidal cell body layer in CA1. In medial PFC, the stimulating electrode was located ~150 μm from the cell soma. Testing stimuli (0.1 ms in duration) were delivered at a frequency of 0.1 Hz to evoke synaptic transmission. NMDAR-mediated EPSCs were pharmacological isolated by blockade of AMPA receptors with bath-applied CNQX (10 μM). Bicuculline (10 μM) was included in the bath to block GABARR_A_RR receptor–mediated transmission. We amplified raw data using a MultiClamp 700B amplifier and a Digidata 1322A acquisition system sampled at 10 KHz and analyzed the data with Clampfit 10.6 (Axon Instruments). Statistical analysis was conducted using SigmaPlot software version 12. The tests used and *P* values are reported in the respective figure legends.

### Surgery for in vivo experiments.

Mice (8–18 weeks of age) were anesthetized with 1% isoflurane and implanted with a low-impedance (<5 kΩ, 1,000 Hz) stainless steel tripolar electrode (PlasticsOne, Roanoke, VA). Electrodes were cut to a length of 3 mm (positive) and 1 mm (ground and reference) and placed at 1 mm intervals along the sagittal axis. The positive electrode was positioned 1.8 mm posterior, 2.65 mm right lateral, and 2.75 mm deep relative to bregma as previously described (54–56). A cannula (Alzet Brain Infusion Kit #3, Durect Corporation, Cupertino, CA) was placed 1 mm left lateral and 0.5 mm posteriorly relative to bregma. The electrode and cannula were secured to the skull with dental cement (Ortho Jet; Lang Dental, Wheeling, IL) and ethyl cyanoacrylate (Elmers, Columbus, OH). The catheter connected to the base of the cannula was driven s.c. on the dorsal surface and the implant was closed with a single stitch (57). Supplemental warmth was provided after surgery with the heating pad. A micro-osmotic pump (Alzet Model #1002, Durect Corporation, Cupertino, CA) was primed for a 48-hour period at 37°C with TAT-SAPIP peptide dissolved in vehicle to achieve a final dose of 1 μg/kg/day. Osmotic pumps were implanted for 14 days. Animals were monitored throughout the course of the study for weight loss, signs of distress, and adverse reactions at the implant site.

### Deviance detection.

Mice were exposed to a series of 19 repetitive standard tones (6 or 9 KHz, 85 dB) followed by a novel tone (9 or 6 KHz, 85 dB) using a flip/flop control procedure. Waveforms were corrected relative to baseline at stimulus onset.

### Analysis.

Only the standard tone presented immediately before the novel tone was used for analysis to ensure that an equal number of standard and novel tones were used. Subtraction of the standard from the novel produced a difference wave, which was quantified by the peak negative value within the relevant time period of 30–80 msec after stimulus for MMN and 60 to 200 msec for the P3A.

### TFC.

TFC took place in rectangular chambers measuring 40 cm long, 15 cm wide × 22 cm high (Med Associates, Fairfax, VA). Training consisted of 3 sets of CS and US pairings. The CS consisted of a 20-second tone (2,700 Hz, 85 dB) while the US was a 2-second 0.6 mA foot shock. The CS and US were separated by a period of 20 seconds (trace period). CS-US pairings were presented at 3, 7, and 11 minutes. Twenty-four hours after training, mice were tested for memory of conditioning. To control for possible influence from contextual learning, the conditioning chambers were altered by placing a thin Plexiglas panel on the floor grid and placing an odorant underneath the floor. The CS was again presented at 3, 7, and 11 minutes, during which time freezing was measured (cue). Freezing was also measured 20 seconds prior to CS onset (precue). Freezing behavior was scored in real time digitally (FreezeScan 1.0, Clever Sys Inc.) and was quantified as percent time freezing.

### Statistics.

No data were transformed, recoded, rescaled, or normalized.

All in vitro data were analyzed via ANOVA or *t* test. In vivo experiments were analyzed via repeated measures ANOVA with time spent freezing during the cue versus precue periods as the repeated dependent variable and with sex, genotype, and treatment as independent variables. The P3a/MMN data were analyzed with a 3-way ANOVA. The TFC data were analyzed with a 3-way repeated-measures ANOVA. Further analyses were conducted with these data that assessed whether the difference between precue and cue (cue minus precue) for each animal was significantly different from zero (i.e., no difference between the amount of freezing between the precue and cue). Post hoc tests were conducted using planned comparison *t* tests. To control for family-wise error when multiple comparisons were needed the Holm-Šidák test was used (52). In all analyses, α was set at *P* < 0.05. All statistical tests were 2 tailed.

### Study approval.

All procedures conducted at the Hospital for Sick Children were performed in accordance the Canadian Council on Animal Care and were approved by the Animal Care Committee of the Hospital for Sick Children (protocol no. 1000065956). All procedures related to in vivo testing were performed at the University of Southern California in accordance with the *Guide for the Care and Use of Laboratory Animals* (National Academies Press, 2011) and approved by the IACUC at the University of Southern California (protocol no. 20646). Postmortem experiments were approved by the IRB at the University of Pennsylvania. Patients were prospectively diagnosed by DSM criteria, and consents for autopsy were obtained from the next of kin or a legal guardian.

### Data availability.

Values for all data points in graphs are reported in the Supporting Data Values file. Otherwise, all data will be made available upon reasonable request.

## Author contributions

REF, SJS, MWS, HL, CGH, KEBW, ASS, and OM designed research studies. REF, OM, HL, PK, AM, MH, ADM, and AT conducted experiments. REF, AB, OM, RLG, HL, PK, MH, ADM, WZ, and AT acquired data. REF, SJS, LMC, FA, KP, HL, PK, AM, MH, ADM, and RJG analyzed data. REF, HL, SJS, MWS, CGH, KEBW, FA, LMC, and RLG provided reagents and wrote the manuscript. SJS, MWS, and CGH oversaw the project.

## Funding support

This work is the result of NIH funding, in whole or in part, and is subject to the NIH Public Access Policy. Through acceptance of this federal funding, the NIH has been given a right to make the work publicly available in PubMed Central.

- R01 MH075916-05 (CGH/SJS)
- RO1 MH116463 & RO1 MH138995 (CGH)
- Gift from Isaac Larian and Family to support psychosis research at USC

## Supplementary Material

Supplemental data

Unedited blot and gel images

Supporting data values

## Version 1 12/04/2025

In-Press Preview

## Version 2 01/23/2026

Electronic publication
