# Supplementary material for: Protein-protein interaction–interfering peptide rescues dysregulated NMDA receptor signaling
Source: JCI Insight. 2025 Dec 4;11(2):e189634. doi: 10.1172/jci.insight.189634 (PMC12892891; doi:10.1172/jci.insight.189634)
Supplement: Supplemental data [file jciinsight-11-189634-s289.pdf]

Supplementary Data

1. Intracellular administration of the Src activator peptide EPQ(pY)EEIPIA potentiates NMDAR EPSCs, but intracellularly applying the Src inhibitor Src40-58 inhibits NMDAR EPSCs in medial PFC (mPFC) of wild type mice. The results presented in Supplementary Figure 1 demonstrate that synaptic NMDAR currents in layer 5 pyramidal neurons in the mPFC are regulated by Src kinase. Data represent mean ± SEM.

2. TAT-SAPIP peptide application does not change baseline synaptic transmission. To investigate whether enhancement of synaptic NMDAR current by SAPIP peptide would induce potentiation of synaptic strength, we monitored baseline synaptic transmission by whole-cell recording EPSPs at CA3-CA1 synapses while applying SAPIP peptide intracellularly. We found that TAT-SAPIP peptide intracellular application did not change baseline synaptic transmission (Suppl Figure 1A). This effect was also confirmed by the observation that CA3-CA1 field EPSPs did not change by bath application of SAPIP peptide up to 60 min (Suppl Figure 1B). Notably, TAT-SAPIP did not affect the current (I)-voltage (V) relationships of NMDARs suggesting that the effects of TAT-SAPI is not via NMDAR gating (Suppl Figure 1C). All measurements were taken from distinct samples. Data represent mean ± SEM.

3. Src is associated with PSD-95 in the synaptoneurosomes but not in the cytosol. Given the oncogenic potential of Src, our strategy is not to enhance src activity globally, but to reduce inhibitory effects of PSD-95 by reducing the Src-PSD-95 association. Its specificity to the synapse is predicated on the fact that Src is not associated with PSD-95 outside the synapse as shown in Supplementary Figure 2. Data represent mean ± SEM.

4. TAT-SAPIP increases Src activity in synaptoneurosomes derived from DLPFC of patients and controls. The results presented in Figure 3D is shown for subjects with schizophrenia and controls separately (Supplementary Figure 3). Data represent mean ± SEM.

Supplementary Figures

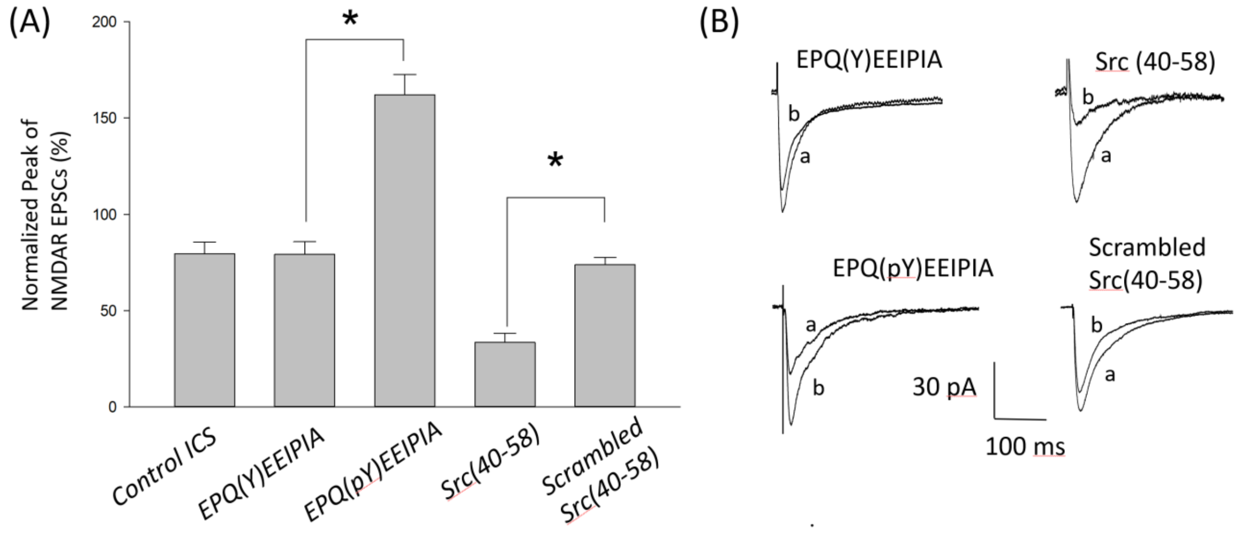

Supplementary Figure 1. Intracellular administration of the Src activator peptide EPQ(pY)EEIPIA potentiates NMDAR EPSCs, but intracellularly applying the Src inhibitor Src40-58 inhibits NMDAR EPSCs in mPFC of wild type mice. (A) Histogram of normalized NMDAR EPSC amplitude for each group at the time of 20 minutes after break-through of whole-cell recording of layer 5 pyramidal neurons in mPFC slices. Intracellularly administrating the Src activator peptide EPQ(pY)EEIPIA (2mM) through a patch pipette significantly potentiates NMDAR EPSC amplitude (159 ± 19 %, n = 7) compared to the non-phosphorylated, inactive form EPQ(Y)EEIPIA (77.0 ± 8.3%, n = 6, p = 0.004). In contrast, intracellularly applying the Src inhibitor Src40-58 (0.03mg/ml) significantly inhibited NMDAR EPSCs (40.7 ± 4.2%, n = 14) compared to the scrambled peptide (73.9 ± 4.2%, n = 8, p =< 0.001). No differences were observed between inactive form EPQYEEIPIA, scrambled Src (40-58) peptide versus recording with only control intracellular solution (ICS). . * $p<0.05$. A one-way ANOVA followed by Holm-Sidak post hoc test was used for statistic comparisons. (B) Representative individual NMDAR EPSC traces from individual mPFC neurons. The traces labelled ‘a’ were taken 2 minutes after breakthrough, and those labelled ‘b’ were taken 20 minutes after breakthrough.

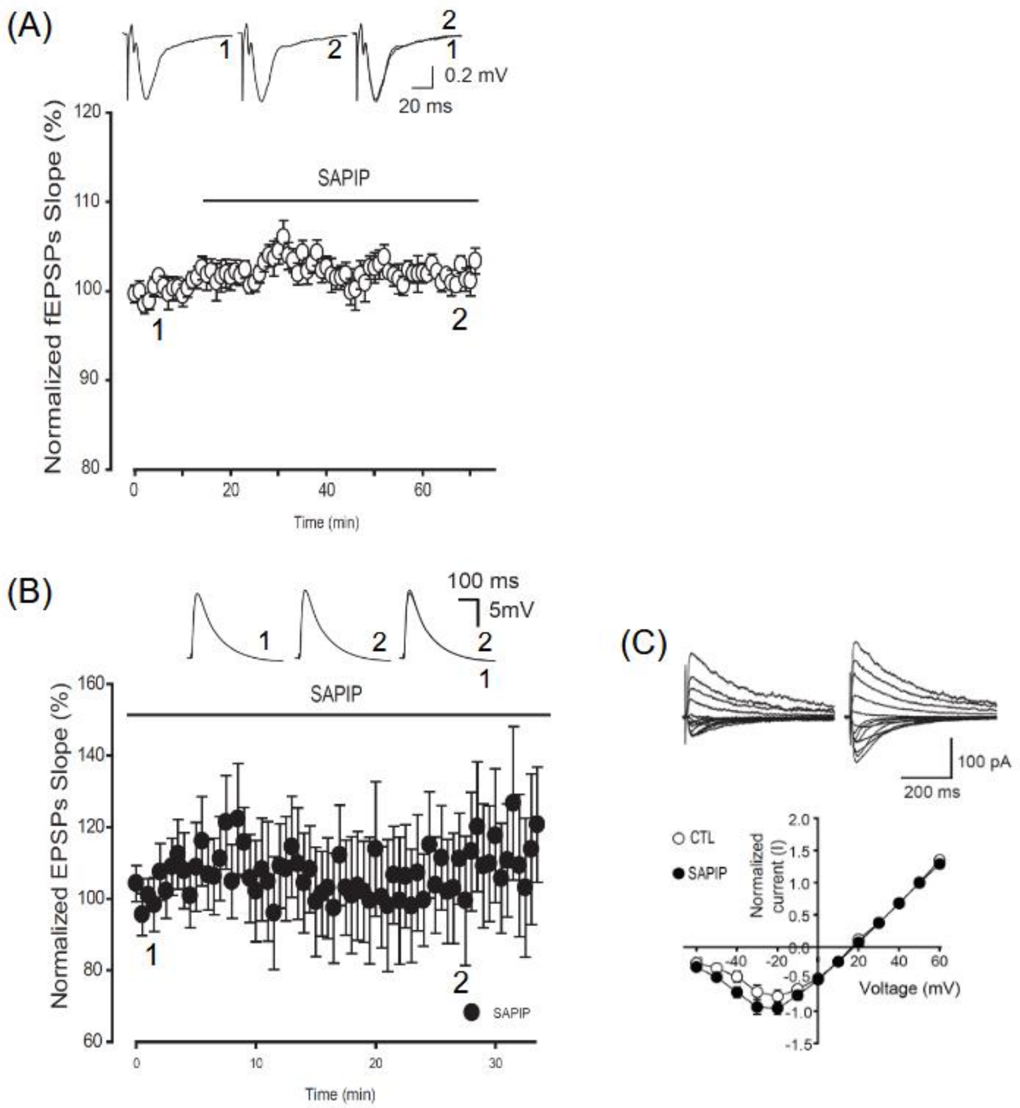

Supplementary Figure 2. SAPIP peptide application does not change baseline synaptic transmission. (A) Scatter plot of NMDAR EPSC peak amplitude over time recorded from CA1 pyramidal neurons taken from wild type mice with intracellularly applied TAT-SAPIP peptide. We monitored baseline synaptic transmission by whole-cell recording EPSPs at CA3-CA1 synapses while applying SAPIP peptide intracellularly. (B) Scatter plot of field EPSPs 0ver time recorded from CA1 pyramidal neurons taken from wild type mice with intracellularly applied TAT-SAPIP peptide.(C) Scatter plot with representative traces showing the current (I)-voltage (V) relationship and reversal potential of NMDAR EPSCs at the end of each recording in panel A. All measurements were taken from distinct samples.

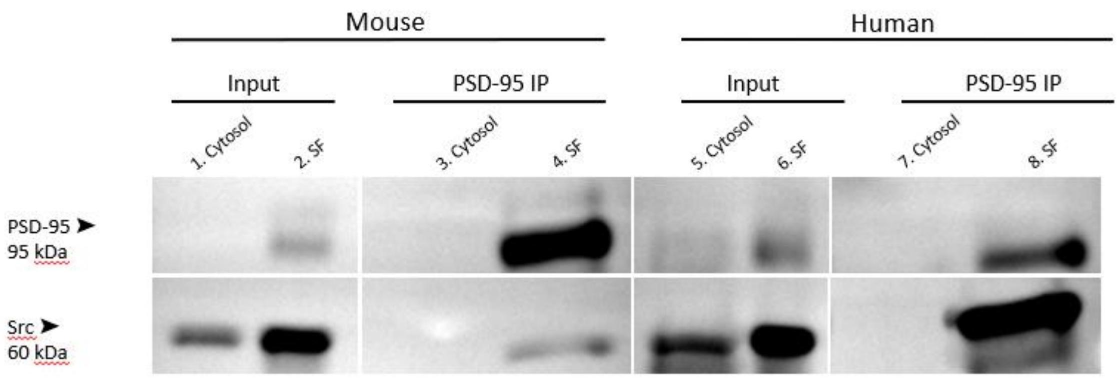

Supplementary Figure 3. Src is associated with PSD-95 in the synaptoneurosomes but not in the cytosol. (Input) Ten micrograms of the cytosol or synaptoneurosomes (SF) from mouse or human cortex were analyzed by Western blot for PSD-95 and Src. PSD-95 was undetectable in the cytosol. (PSD-95 IP) 100 micrograms of cytosol and 50 micrograms of synaptoneurosomes derived from mouse or human cortex were IPed for PSD-95 and probed for Src. The Src-PSD-95 association was seen only in the synaptoneurosomes.

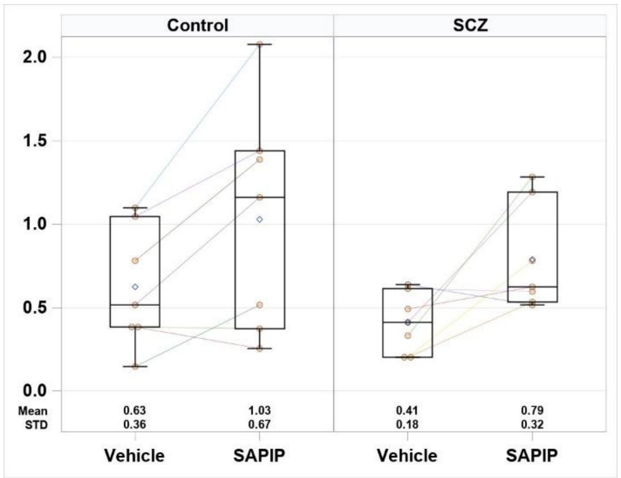

Pairwise comparisons						
**Contrast**	**Dx**	**Treatment**	**Num DF**	**Den DF**	**F**	**Pr > F**
CONTROL vs SCZ		Vehicle	1	12	0.87	0.3705
Control vs SCZ		SAPIP	1	12	1.14	0.3067
Vehicle vs SAPIP	CTRL		1	12	7.39	0.0187
Vehicle vs SAPIP	SCZ		1	12	6.31	0.0273

Supplementary Figure 4. TAT-SAPIP increases Src activity in synaptoneurosomes derived from patients and controls. To test if TAT-SAPIP modulates Src activity differentially between control vs. schizophrenia subjects, the results represented in Figure 3D were analyzed by a two-way repeated measures ANOVA. A between subject factor was defined as the condition (or diagnosis) (Control vs. Schizophrenia) and a within subject measure was set as the treatment (Vehicle vs. SAPIP). This model allowed us to account for the within-subject correlation (Vehicle vs. SAPIP) with r=0.5597. We do not see a diagnosis by treatment interaction (F(1,12)=0.02, p=0.89) or a diagnosis main effect (F(1,12)=1.28, p=0.28). We do however see a significant treatment effect (F(1,12)=13.68, p=0.003) which indicates that SAPIP increases Src activity in both groups (Supplementary Figure 4A). Moreover, we made pairwise contrasts between the 4 cells (Supplementary Figure 4 B). The first two contrasts focus on between group differences in either Vehicle or SAPIP. The last two contrasts focus on the effects of SAPIP in each group. SAPIP induced significant differences in both the control and schizophrenia groups (P=0.0187, P=0.0273 respectively).

Supplementary Table 1. Demographic Characteristics

ID	Sex	Age	PMI	Rx	pH	CPZ (mg/Day)	Antipsychotic	Race
1	F	74	3.5	N	6.62	N/A		C
2	F	76	9.5	S	6.52	35	quetiapine	C
3	F	89	7	N	6.3	N/A		AA
4	F	76	9	S	6.71	303	risperidone	C
5	F	92	5	N	6.5	N/A		C
6	F	88	7.5	S	6.58	UnK	UnK	C
7	F	90	6	N	5.98	N/A		C
8	F	95	8.5	S	6.77	0	none	C
9	M	86	7	N	6.36	N/A		C
10	M	82	19.5	S	6.54	815	haloperidol	C
11	M	98	15	N	6.22	N/A		C
12	M	89	15	S	6.42	102	thiothixene	C
13	M	69	11	N	6.49	N/A		C
14	M	81	9	S	6.19	158	olanzapine	C
