# Supplementary figures and images for: Protein-protein interaction–interfering peptide rescues dysregulated NMDA receptor signaling
Source: JCI Insight. 2025 Dec 4;11(2):e189634. doi: 10.1172/jci.insight.189634 (PMC12892891; doi:10.1172/jci.insight.189634)

Figure 1B

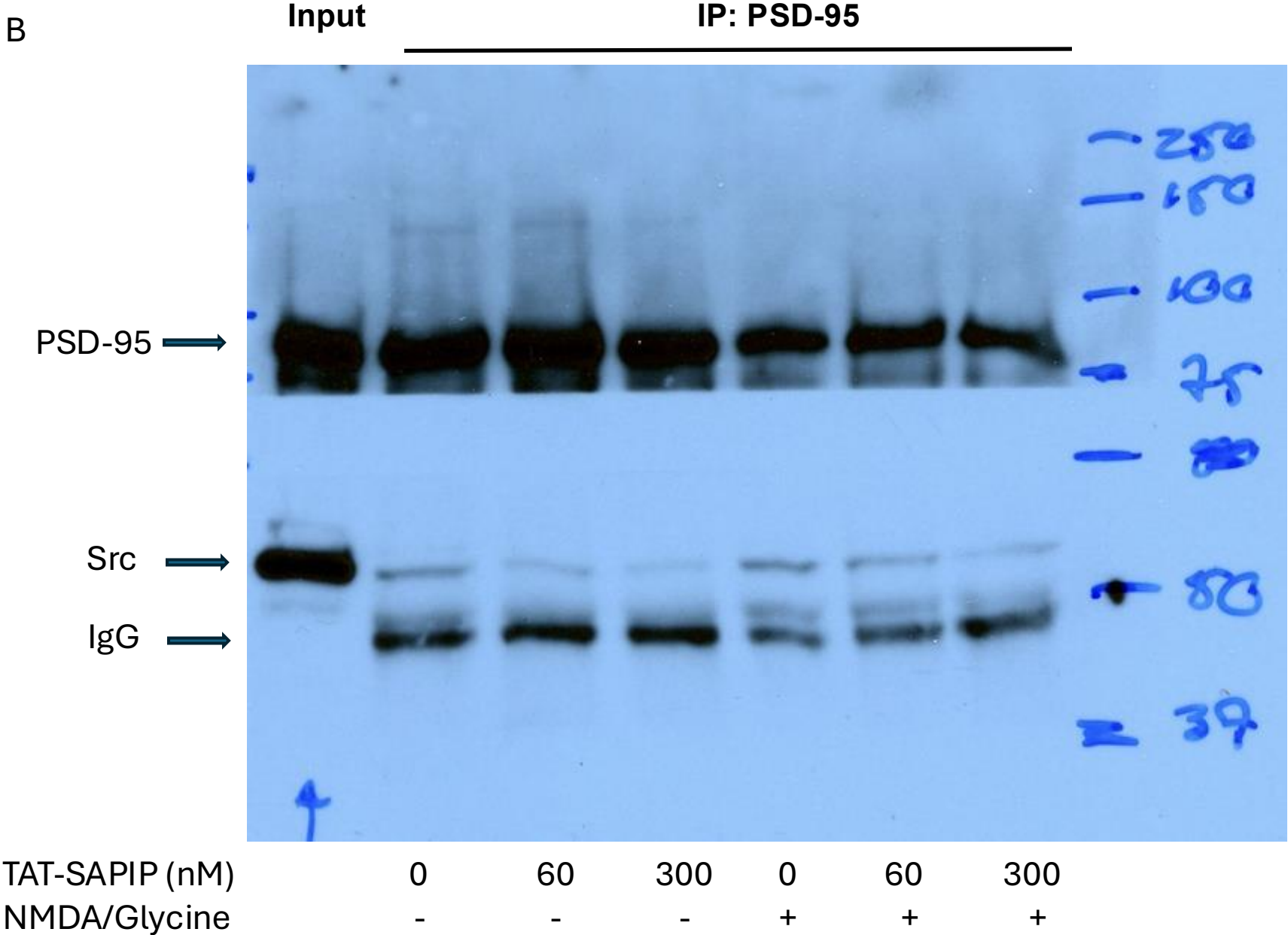

Supplement: Unedited blot and gel images [file jciinsight-11-189634-s290.pdf]
